# Roles, detection, and visualization of neutrophil extracellular traps in acute pancreatitis

**DOI:** 10.3389/fimmu.2022.974821

**Published:** 2022-08-05

**Authors:** Hongxuan Li, Lingyu Zhao, Yueying Wang, Meng-Chun Zhang, Cong Qiao

**Affiliations:** ^1^ Department of General Surgery, The First Affiliated Hospital of Harbin Medical University, Harbin, China; ^2^ Department of Pathology, Harbin Medical University, Harbin, China; ^3^ Department of Pathology, The First Affiliated Hospital of Harbin Medical University, Harbin, China; ^4^ Department of Pharmacology, Harbin Medical University, Harbin, China

**Keywords:** neutrophil extracellular traps, acute pancreatitis, multicolor immunofluorescence, citrullinated histone 3, peptidylarginine deiminase 4

## Abstract

Neutrophil extracellular traps (NETs) are produced in large quantities at the site of inflammation, and they locally capture and eliminate various pathogens. Thus, NETs quickly control the infection of pathogens in the body and play vital roles in immunity and antibacterial effects. However, evidence is accumulating that NET formation can exacerbate pancreatic tissue damage during acute pancreatitis (AP). In this review, we describe the research progress on NETs in AP and discuss the possibility of NETs as potential therapeutic targets. In addition, since the current detection and visualization methods of NET formation are not uniform and the selection of markers is still controversial, a synopsis of these issues is provided in this review.

## Introduction

Acute pancreatitis (AP) begins with cellular injury and endoplasmic reticulum stress due to premature activation of digestive enzymes in acinar cells (1–5). The associated processes include converting trypsinogen to trypsin by lysosomal hydrolase cathepsin B (3, 6) and then degrading trypsin by cathepsin L (6–8). Subsequently, protease activation cascade induces cell death, releasing danger molecules known as damage-associated molecular patterns (DAMPs), and eventually activating the immune system (3, 7). In this process, the intensity of the immune response determines the possibility of systemic complications and disease severity (9–11). After the onset of AP, neutrophils are the first set of leukocytes that infiltrate the pancreatic tissue, directly inducing the activation of intracellular proteases, promoting necrotic cell death, and occluding the acinar lumen leading to pancreatic damage (9, 12–15). An avalanche of research reports indicates that the aggregation of neutrophils in pancreatic tissues is a key factor in the development of AP. Mechanistically, this is because neutrophils infiltrating the pancreatic tissue can aggravate tissue damage by releasing reactive oxygen species (ROS) (13), enzymes (such as elastase and matrix metalloproteinase-9) (16, 17) and tumor necrosis factor-α (TNFα) (12, 18).

There is growing evidence that the web-like structures released by neutrophils, the neutrophil extracellular traps (NETs), can promote pancreatic tissue damage in AP (19–21). However, presently, the available detection and visualization methods of NET formation are not uniform. Due to the different stimulation of neutrophils, histone H3 may not be citrullinated during the NET formation, so the use of citrullinated histone H3 (citH3) or peptidylarginine deiminase 4 (PAD4) as a marker for NETs remains controversial (22, 23). In addition, given the diversity of AP models, the effect of different stimuli on markers may also be one of the controversial factors. Therefore, in this article, we review the research progress between NETs and AP along with the detection and visualization methods, and selection of markers underlying NET formation.

## Mechanisms of NET formation

NETs are web-like structures with decondensed chromatin fragments as the skeleton and wrapped in histones, proteases, granules, and cytoplasmic proteins (24). Based on recommendations published by experts in this field, the term “NET formation” is used to describe the process by which neutrophils produce and release NETs (25).Currently, there are two known pathways for NET formation. The first is the cell death pathway known as lytic NET formation, which begins with nuclear delobulation and the disassembly of the nuclear envelope and continues until the loss of cell polarization, chromatin decondensation, and plasma membrane rupture. The second is non-lytic NET formation, which can occur independently of cell death and involves the secretory excretion of nuclear chromatin that is accompanied by the release of granular proteins through degranulation (26). According to the literature, non-lytic NET formation occurs within minutes of exposure to *Staphylococcus aureus*, during which there is no cell death (27, 28). In addition to this, P-selectin/P-selectin glycoprotein ligand-1 mediates neutrophil-platelet interaction (29, 30) and activated platelets can promote non-lytic NET formation through high mobility group protein B1 (HMGB1)/receptor for advanced glycation end products (31). In view of the peculiarity of the non-lytic NET formation, most of the current studies are conducted in lytic NET formation background.

In the NET formation pathway, the process from ROS generation to chromatin decondensation is the core mechanism. However, it is noteworthy that the inhibition of leukocyte signal inhibitory receptor 1 prevented NET formation without affecting ROS (32). Due to various stimuli, calcium ions are released from the endoplasmic reticulum into the cytoplasm, resulting in an increase in the production of ROS through the NADPH oxidase complex, which in turn activates the protein kinase C or RAF-MEK-MAPK pathway, resulting in NET formation (23, 33, 34). In the aforementioned process, one of the most important ways is to stimulate myeloperoxidase (MPO) through the generated ROS to activate neutrophil elastase (NE) and facilitate the transfer of NE from the azurophilic granules to the nucleus. NE transferred to the nucleus can destroy chromatin packaging by hydrolyzing histones, and then MPO binds to chromatin and synergizes with NE to cause chromatin decondensation (35). However, one study found that inhibiting the enzymatic activity of MPO only delayed NET formation, but did not prevent NET formation (36). NE plays a more important role in this process because NE needs to bind to F-actin filaments in the cytoplasm and degrade them before entering the nucleus to drive chromatin decondensation (37). In addition, the results of the *in vitro* studies showed that NE is sufficient to disintegrate the nucleus (35). Therefore, in the MPO-NE pathway, compared with MPO, the effect of NE activity on NET formation may be more important. In addition to the MPO-NE pathway, another well-studied pathway related to chromatin decondensation is PAD4-driven histone citrullination (38, 39). It has been found that the activation of PAD4 requires a reducing environment (40), but the inhibition of NADPH oxidase still reduces the occurrence of PAD4-driven histone citrullination (24, 26). This is because hydrogen peroxide and ROS generated by NADPH oxidase activation are sufficient to activate PAD4 (41–43). However, ROS production was not eliminated by the inhibition of NADPH oxidase, possibly due to increased mitochondrial ROS production (44). In addition, NADPH oxidase is negatively regulated by active PAD4 (45).

## Role of NETs in acute pancreatitis

**Figure 1:**
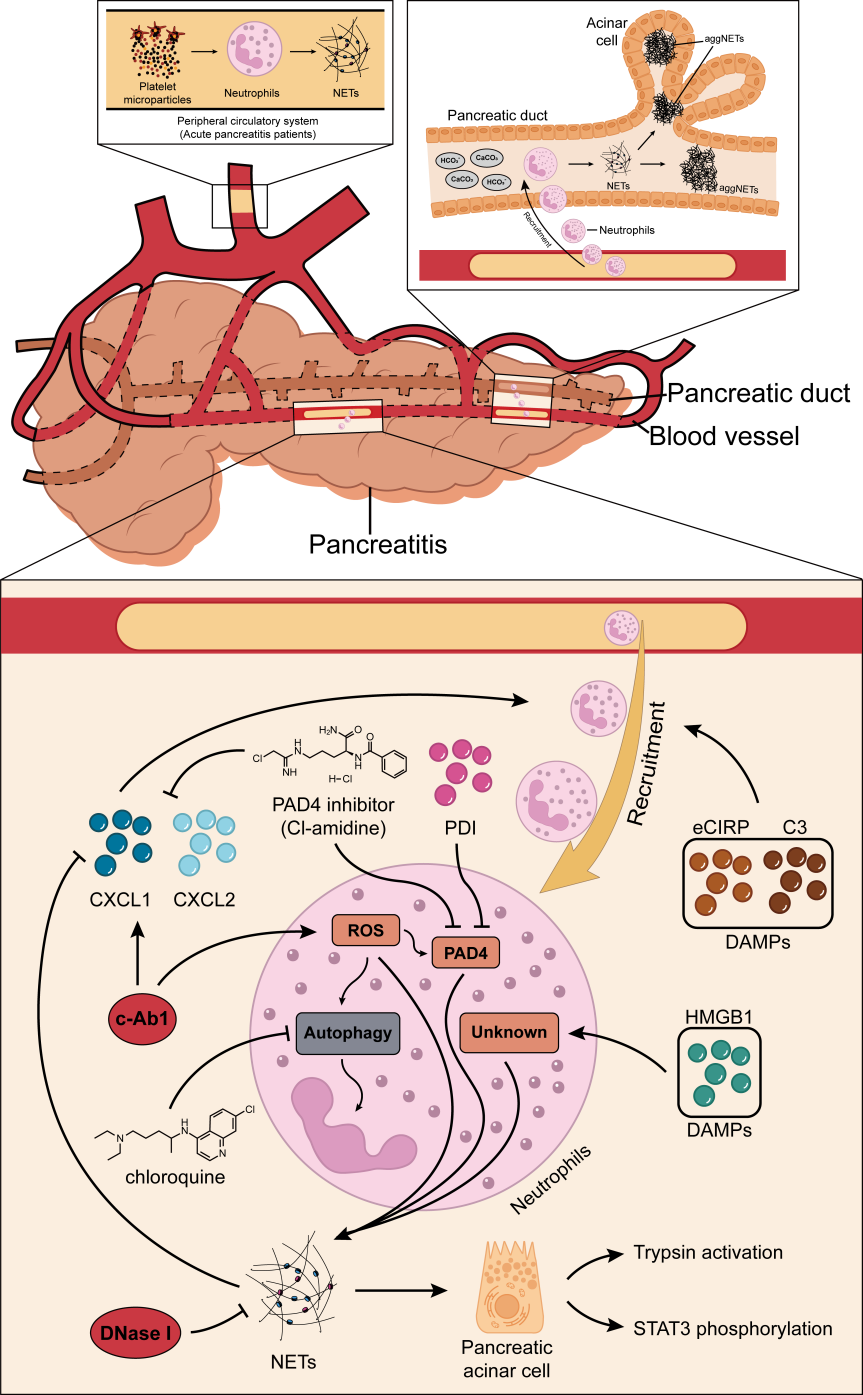
Roles of NETs in AP. NETs may inherently function as chemotactic signals to induce the recruitment of neutrophils, or they may stimulate the release of related chemokines (CXCL1 and CXCL2). c-Abl kinase, eCIRP, HMGB1, protectin D1, and complement C3 promote NET formation through the PAD4-driven histone citrullination pathway. Autophagy is also thought to play a role in the NET formation in AP. IL-17A promotes the accumulation of neutrophils in the pancreatic duct, and the bicarbonate ions and calcium carbonate crystals in the pancreatic juice will then stimulate the accumulated neutrophils to generate aggregated NETs and these can occlude the pancreatic duct, inducing pancreatitis. In addition, platelet microparticles can significantly promote NET formation. NETs, neutrophil extracellular traps; AP, acute pancreatitis; CXCL1, C-X-C motif chemokine ligand 1; CXCL2, C-X-C motif chemokine ligand 2; eCIRP, extracellular cold-inducible RNA-binding protein; HMGB1, high mobility group protein 1; PAD4, peptidylarginine deiminase 4.

Many recent studies have shown that NETs may play a pivotal role in the development of AP (19–21). Elimination of NET formation by injecting DNase I into mice effectively reduced CXCL2 production and neutrophil recruitment in inflamed pancreatic tissue (19). The results showed that NETs themselves may play a role as chemotactic signals, or NETs stimulated the release of related chemokines. In addition, the results of the *in vitro* studies showed that histones (histones 2A, 2B, 3, and 4) in NETs can regulate STAT3 activity and trypsin activation in acinar cells, and their effects on acinar cells are similar to cerulein (19). Emphatically, the formation of NETs is partially dependent on the ROS production. For instance, a study found that c-Abl kinase can promote ROS production and NET formation. This process is accompanied by the increased expression of citH3. Concurrently, inhibition of c-Abl kinase can reduce inflammation and tissue damage in AP (46). ROS can induce autophagy (47) and NET formation is dependent on autophagy (48). Researchers have found that when specific inhibitors for autophagy were injected into mice that NET formation was also inhibited, and this was due to the expression of PAD4, and consequently the severity and survival rates for AP were improved (21). Citrullination of histones is usually driven by PAD4, as described previously. Therefore, a study demonstrated the role of PAD4 in reducing NET formation in pancreatic tissue of severe acute pancreatitis (SAP) by the oral administration of Cl-amidine, a specific inhibitor of PAD4, and the construction of PAD4 knockout (PAD4−/−) mice (21), respectively. In addition, the inhibition of PAD4 expression reduces pathological inflammation and tissue damage in the inflamed pancreas (20). In fact, a recent study also found that the injection of protectin D1 into mice can effectively inhibit the expression of PAD4, thereby reducing NET formation and improving AP (49). Furthermore, the premise of NET formation is the accumulation of neutrophils in pancreatic tissues, where the release of DAMPs plays an important role. The results of the studies found that extracellular cold-inducible RNA-binding protein (eCIRP) and complement C3 can act as DAMPs to promote neutrophil accumulation in pancreatic tissues, which, in turn, leads to NET formation and pancreatic tissue injuries (50, 51). Concurrently, eCIRP is also a component of NETs and can induce acinar cells to secrete amylase by binding to the TLR4 complex in the acinar cell membrane (50). This result suggests that NETs themselves may function as DAMPs or chemotactic signals to some extent. NETs can also be formed by responding to extracellular HMGB1 and histones in a TLR4- and TLR9-dependent manner (26). The results show that HMGB1 can cause pancreatic tissue injury by activating NET formation, but the specific mechanism of activation is not clear (52). To date, the most comprehensive research has shown that IL-17A promotes the accumulation of neutrophils in the pancreatic duct, and bicarbonate ions and calcium carbonate crystals in the pancreatic juice stimulate the accumulated neutrophils to form aggregated NETs (aggNETs), which can then occlude the pancreatic duct, inducing pancreatitis. Notably, the study also found that no intraductal aggNETs were found in cerulein-induced AP, and disease progression in this experimental animal model was independent of PAD4 (53). Based on the above results, further studies found that both low pH and high carbon dioxide/bicarbonate ratio reduced the ability of neutrophils to release NETs (54). Usually, bicarbonate can effectively increase pH, which can then increase the calcium influx, mitochondrial ROS generation, PAD4 activity, and histone 4 cleavage, thereby promoting NET formation (55, 56). Therefore, the alkaline environment provided by pancreatic juice might provide better mechanistic insight into the etiology of AP. In addition, studies have found that platelet particles in plasma samples from patients with AP can significantly promote NET formation, and the level of platelet particles is positively correlated with the severity of AP (57). In summary, reducing NET formation is an effective strategy to improve pancreatic tissue injury in AP, but the relevant mechanisms need to be further explored. **Figure 1** summarizes the potential mechanisms of NET formation in AP.

## Detection and visualization of NETs in pancreatic tissues of AP

Numerous studies have reported using immunofluorescence staining is often used as a method to detect NET formation in tissue samples (58–60). This is because NET formation is characterized by the co-localization of extracellular DNA, nuclear proteins and granular (or cytoplasmic) proteins, which are significantly separated and relatively fixed from the nucleus in resting neutrophils (61). One study tested diverse antigen retrieval methods and various combinations of commercially available antibodies, and it was found that NETs in the tissue could be best detected when using a mild antigen retrieval protocol and a combination of the NE and histone H3 antibodies (62). In existing studies, the available detection and visualization methods of NET formation in pancreatic tissues of AP are not consistent. In some studies, researchers used scanning and transmission electron microscopies combined with immuno-double-gold labeling technique to detect NET formation, using citH3 or histone2B as the marker of NETs and elastase as a marker of neutrophils (19, 20, 50, 51). Because it is difficult to identify neutrophils from cell morphology by scanning electron microscopy, transmission electron microscopy combined with immuno-double-gold labeling technique can make up for the deficiency of the former. The advantage of this technique is that the pretreatment steps have little effect on the microstructure, and the gold particles have high electron density, which make them clearly distinguishable from other immune products under the electron microscope. In addition, some researchers use immunofluorescence staining to detect NET formation. In this process, they chose citH3 as the marker of NETs, MPO or Ly-6G as the marker of neutrophils (21, 49), or directly chose SYTOX Green dye-labeled extracellular DNA as the marker of NET formation (52). In view of the fact that most of the aforementioned studies focused on the histone citrullination in chromatin driven by PAD4, which depolymerizes the chromatin and promotes NET formation, it is reliable to use citH3 as a marker in the detection of NET formation. However, considering the different stimuli in the construction of AP mouse model, we cannot rule out the possibility that NETs are produced by other ways or histone citrullination driven by PAD4 is not the main pathway; thus, if citH3 is used as the sole marker, it may lead to a potential deviation. Therefore, further studies may be needed to discuss the selection of NETs-related markers and the relationship between NETs and AP. A summary of the advantages and disadvantages of each technique based on the corresponding AP mouse model is provided in **Table 1**.

**Table 1:** Detection and visualization of NETs in pancreatic tissues.

AP models	Detection method	Marker selection	Advantages	Possible problems
Taurocholate (19, 20, 50, 51)	Transmission electron microscopy **+** Immuno-double-gold labeling	NET-marker: citH3 or Histone 2B Neutrophils-marker: Elastase	The preprocessing steps have less effect on the microstructure; The gold particles have a high electron density, which are clearly distinguishable from other immune products under the electron microscope.	citH3 independent pathways are not detected.
Taurocholate (46); Careulein (52)	Western Blot	NET-marker: citH3 or PAD4		Do not provide the required subcellular resolution; Do not readily allow simultaneous localization of two antigens.
Taurocholate (52); L-arginine (19)	SYTOX Green staining	NET-marker: Extracellular DNA		Poor stability and toxicity; Cannot differentiate between necrotic and NET-cells.
Careulein (49); Pancreatic duct ligation (49); L-arginine (21)	Immunofluorescence staining	NET-marker: citH3 Neutrophils-marker: MPO or Ly-6G	Provide the required subcellular resolution; Allow simultaneous localization of two antigens.	citH3 independent pathways are not detected.

NETs, neutrophil extracellular traps; AP, acute pancreatitis; citH3, citrullinated histone H3; PAD4, peptidylarginine deiminase 4; MPO, myeloperoxidase; Ly-6G, lymphocyte antigen 6 complex locus G6D.

## Discussion

An overwhelming number of studies have found that NETs can contribute to inflammation and injury of organs in mice with AP, and thus may be used as a target to reduce pancreatic tissue injuries and inflammation in patients with AP. However, there are a few issues that deserve further discussion, as outlined below. First, are there any other pathways, aside from PAD4-driven histone citrullination pathway, that are involved in the occurrence and development of AP? If so, which one is the main pathway? For now, this issue remains open for exploration in future studies. Second, most studies, including AP, used citH3 as the sole marker of NETs (59, 63), but the use of citH3 or PAD4 as a marker of NETs is controversial (22, 23). Although the detection of citH3 is considered a minimum requirement for the identification of NET formation, quantification biases caused by citH3-independent pathways should also be considered. To address this, co-staining analysis of multiple pathway-related markers should be conducted to visualize NET formation in pancreatic tissues. The components of NETs include MPO and NE: in the resting neutrophils, they do coexist in granules, and their localization with the nucleus is relatively fixed. However, after NET formation, due to chromatin depolymerization and nuclear membrane rupture, the positions of MPO, NE and nuclear DNA become unclear, and the original relatively fixed positions are dismantled, resulting in residual mixing of nucleus, cytoplasm, and granules, which provides conditions for the co-localization of the three. Therefore, a comprehensive visual qualitative analysis of co-staining of MPO, NE and citH3 by immunofluorescence may provide a more comprehensive assessment of NET formation, but further research and discussion are needed in quantitative aspects. Furthermore, it is worth noting that while NET-enriched area can be quite spacious, NETs can be significantly less stretched and contain only a few neutrophils in dense tissue, as in myocarditis (61, 64). Therefore, it is crucial to show the colocalization of nuclear and granular components more clearly by immunofluorescence staining. With the advent of multiple fluorescence immunohistochemicals, this issue can be successfully addressed. A third issue is that of generalizability. Invasive interventions are rarely performed in the early stages of SAP and the optimal timing of invasive interventions is still unclear (65), making it difficult to obtain human pancreatic tissue samples. Currently, only blood samples from patients can be tested for NET formation, and there is still a lack of effective detection methods of NET formation in human pancreatic tissue samples. This limitation is difficult to overcome. Finally, as a fourth issue, we want to remark on an interesting outlook. The premise of NET formation in AP is the accumulation of neutrophils in pancreatic tissues, it is also possible that there are neutrophil subsets with different molecular signatures during pancreatitis. Furthermore, these subsets may respond differently to environmental challenges that subsequently affect their polarization and activation. With the advent of single-cell sequencing technology, combined with analyses based on the technology of cytometry by time-of-flight (CyTOF) mass spectrometry (66, 67), the heterogeneity of neutrophils can be better elucidated. At the same time, this could also provide more directions for follow-up studies on neutrophils that produce NETs with different phenotypes and functions. Taken together, the related research progress is still limited, although these data suggest that NETs have a therapeutic potential in AP.

## Author contributions

HL and CQ developed the concept and design. HL and LZ analyzed the data. HL and CQ wrote the manuscript. LZ, YW, M-CZ, and CQ provided critical discussion, supervised the study, and edited the manuscript. All authors reviewed the paper. All authors contributed to the article and approved the submitted version.

## Funding

This work was supported by a grant from the Innovation Foundation of Higher Education of Heilongjiang Province (Grant No.900204).

## Acknowledgments

We would like to thank Editage (www.editage.cn) for English language editing.

## Conflict of interest

The authors declare that the research was conducted in the absence of any commercial or financial relationships that could be construed as a potential conflict of interest.

## Publisher’s note

All claims expressed in this article are solely those of the authors and do not necessarily represent those of their affiliated organizations, or those of the publisher, the editors and the reviewers. Any product that may be evaluated in this article, or claim that may be made by its manufacturer, is not guaranteed or endorsed by the publisher.
